# Aquatic and terrestrial heart rates in fur seals: evidence for delayed metabolic processing

**DOI:** 10.3389/fphys.2026.1755942

**Published:** 2026-04-30

**Authors:** Melissa J. Walker, Daniel P. Costa, Stephen P. Kirkman, Andrew J. Hoskins, W. Herman Oosthuizen, Deon Kotze, Michael A. Fedak, John P. Y. Arnould

**Affiliations:** 1 School of Life and Environmental Sciences, Deakin University, Burwood, VIC, Australia; 2 Scripps Institution of Oceanography, University of California, San Diego, San Diego, CA, United States; 3 Department of Forestry, Fisheries and the Environment: Oceans and Coasts, Cape Town, South Africa; 4 Institute for Coastal and Marine Research, Nelson Mandela University, Gqeberha, South Africa; 5 North Australian Indigenous Land and Sea Management Alliance, Brinkin, NT, Australia; 6 School of the Environment, University of Queensland, Brisbane, QLD, Australia; 7 James Cook University, Townsville, QLD, Australia; 8 Oceans and Coastal Research, Department of Forestry, Fisheries and the Environment, Cape Town, South Africa; 9 Sea Mammal Research Unit, Scottish Oceans Institute, University of St Andrews, St Andrews, United Kingdom

**Keywords:** foraging, haul out, onshore, otariid, physiological debt

## Abstract

A seal’s heart rate is affected by their physiological adaptation to aquatic and terrestrial environments. At-sea, heart rate during dive bouts cycles between bradycardia during dives and tachycardia for oxygen replenishment on the sea-surface. While onshore, heart rate reflects apnoea and eupnoea. Intriguingly, complete sea-and-onshore heart rate traces and any relationship between sea and subsequent onshore heart rate, including any immediate or delayed physiological adaptation, remain unexamined. In this study, sea-and-onshore heart rate traces from female Cape fur seals (*Arctocephalus pusillus pusillus*; CFS, *N* = 4) and Australian fur seals (*A. p. doriferus*; AUFS, *N* = 8) revealed expected at-sea cycles of bradycardia (mean minimum beats·min^-1^, CFS: 14.8 ± 1.3 SE; AUFS: 5.7 ± 1.6) and tachycardia (mean maximum beats·min^-1^, CFS: 161.1 ± 1.5; AUFS:163.8 ± 2.6), with interspaced periods where heart rate stabilized as the seal swam at the sea surface. Following haul-out, heart rate traces revealed peaks approximately 20 to 40 beats·min^-^¹ above the apparent minimum, reaching a maximum 6 to 8 hours following the seal’s return to land. Potentially, this onshore heart rate trace reflects a physiological response attributable to delayed compensation for at-sea debt, a scenario explored with multiple linear analyses of area under the heart rate curve, the results of which were all significant (*p* < 0.05) but varied in strength (*R^2^* range from 0.61 to 0.86). These findings underscore the complex interplay of heart rate across aquatic and terrestrial environments, highlighting the benefits of examining holistic physiological traces, and potentially revealing evidence for delayed metabolic processing.

## 1 Introduction

Heart rate profiles offer a powerful lens into how behavioural shifts shape physiological demands in animals. As heart rate closely tracks oxygen consumption, and thus energy expenditure, its patterns reveal how individuals adjust energy allocation across key behavioral and physiological processes in response to natural environments (Green, 2011). Researchers estimate energy expenditure differently in captive and free-ranging animals. In captive animals, energy expenditure can be quantified by calibrating heart rate against measured metabolic rate, while in free-ranging animals, heart rate profiles are interpreted qualitatively to infer relative changes in energy use (Green, 2011). In free-ranging animals, heart rate measurements provide valuable insight into behaviour and physiology under natural conditions and across extended timescales, but when applied without direct metabolic calibration, variability in the heart rate-oxygen consumption relationship can introduce substantial error in energy estimates (Green and Frappell, 2007). Given the constraints of applying the heart rate-oxygen relationship in free-ranging animals, physiological effort must often be approximated from the qualitative method, typically as integrated heart rate over time. This cumulative measure, that can be expressed as the area under the heart rate curve over time (e.g Kovács et al., 2016a, Kovács et al., 2016b), provides a relative index of energetic demand across behavioural states.

Across longer temporal scales, many animals appear to operate in a steady physiological state, yet at finer scales their behaviour is organized into discrete bouts of activity, with phases of feeding, locomotion, and subsequent rest and recovery. Such cyclical patterns are evident even in grazing herbivores (Owen-Smith et al., 2010) and become more pronounced in predators, particularly larger terrestrial carnivores that rely on short bursts of intense exercise to capture prey (Carbone et al., 2007) during which metabolic demand exceeds the capacity of aerobic pathways, resulting in lactate accumulation that must subsequently be cleared (Rabinowitz and Enerbäck, 2020). Air-breathing marine predators that dive for food are even more constrained, foraging in environments devoid of accessible oxygen and returning to the surface to respire. Seabirds and pinnipeds must therefore balance the challenge of acquiring prey without environmental oxygen while also spending time at the surface to replenish oxygen stores and digest meals (Butler and Jones, 1997; Sparling et al., 2007; Thompson and Fedak, 2001). For species provisioning offspring onshore, an additional constraint is the need to maximize energy gain while foraging far from their colony and young, before returning to provision them, with this central-place foraging pattern shaping both foraging decisions and the physiological responses associated with acquisition, digestion, and transfer of energy (Costa, 2007). While analyses at longer temporal scales may obscure short-term fluctuations in oxygen use, focusing only on fine-scale bouts may overlook the accumulation and repayment of physiological costs, particularly in diving animals, for whom oxygen availability and metabolic demand vary across dive-to-surface and at-sea-to-onshore cycles. As oxygen ultimately supports all sustained metabolic activity and the heart governs its delivery with each beat providing a functional “pulse” to the tissues, heart rate provides a practical index of metabolic activity across activity bouts at short and longer-term temporal scales for diving oxygen breathing animals, encompassing diving, surface recovery, and protracted at-sea to onshore energy management.

Pinnipeds (phocid seals, fur seals, sea lions and walruses) manage some physiological costs through cardiac adaptation. For example, within a foraging bout, seals dive repeatedly, with the dive response typified by breathe-holding and significantly reduced heart rate (“bradycardia”) as seals descend the water column, the magnitude of which is dependent on species, dive type, and breath-holding capacity (Elsner et al., 1964; Irving et al., 1941; McDonald and Ponganis, 2014; Scholander, 1940; Thompson and Fedak, 1993). Between the dives are short surface periods, characterized by hyperventilation and elevated heart rate (“tachycardia”); during these periods, seals consume captured prey, while restoring oxygen to tissues, clearing excess carbon dioxide and lactate, and allowing nitrogen absorbed during the preceding dive to diffuse from the blood and tissues, the magnitude of which is greater in otariids that begin dives with larger lung air stores than phocids (Sparling et al., 2007; Williams et al., 2004). Between foraging bouts, pinnipeds spend protracted periods resting at sea. In phocids, such as northern elephant seals, *Mirounga angustirostris*, these rest periods can include ‘drift dives,’ during which seals sink or drift through the water column, a behaviour that is thought to facilitate rest, sleep, and potentially food processing (Mitani et al., 2010). In contrast, such dive types are not commonly described in otariids, which tend remain on the sea surface between dive bouts. These surface intervals may function similar to onshore hauls-outs in that they are used to rest and to support metabolic processes, including digestion (Sparling et al., 2007). Following a foraging trip, seals return to land where they exhibit similar heart rate patterns, alternating between apnoea and eupnoea, which are associated with thermoregulation and development of breath-hold associated with the dive response (De León et al., 2024; Deacon and Arnould, 2009).

Fur seal heart rate has been investigated in past studies, but these studies did not examine a complete and continuous sea-and-onshore cycle. Rather, they have examined heart rate at-sea and onshore in isolation, in some cases, over just a few hours. Collecting short-term and fragmented heart rate data, while important for specific questions, restricts interpretation of heart rate profiles, especially given that the entire sea-and-onshore cycle can last longer than a week. For example, female Cape fur seals, *Arctocephalus pusillus pusillus*, and Australian fur seals, *A. p. doriferus*, typically forage at-sea for ~3.7 to 6.8 days (influenced by season), and then spend ~1.7 to 1.9 days onshore before their next trip to sea (Arnould and Hindell, 2001; Gamel et al., 2005). While many of the most frequently cited foundational studies on heart rate in fur seals gathered data across deployment periods ranging from a few hours to several weeks, these primarily focused on at-sea diving heart rate, and again, often selected narrow periods of time or specific behaviours. When reported at all, descriptions of onshore heart rate profiles from fur seals tend to lack continuous detail, typically reduced to a single average rather than capturing heart rate profiles over time (Boyd et al., 1999; Deacon and Arnould, 2009). Similar average heart rate values are evident in studies on harbour seals, *Phoca vitulina* (Fedak et al., 1988; Williams et al., 1991), Weddell seals, *Leptonychotes weddellii* (Hill et al., 1987), and California sea lions, *Zalophus californianus* (McDonald and Ponganis, 2014) for which only some onshore data are available. Consequently, it is not known whether there is a relationship between heart rate at-sea and heart rate maintained later when seals haul out. The importance of capturing a complete sea-and-onshore cycle is exemplified by a past study by Sparling et al. (2007) whom observed that grey seals, *Halichoerus grypus*, “resting” on the sea-surface exhibit elevated heart rate following dive bouts, an increase linked to the food captured during those dives, and an adaptation that likely allows seals to manage diving demands by delaying digestion until these post-dive surface periods. This raises the possibility that seals could delay digestion and other physiological processes until an even later time, perhaps once they haul out ashore.

The present study investigated heart rate in two seal subspecies which span two distinct foraging modes and differ in average mass: the pelagic-foraging Cape fur seal, *A. p. pusillus*; and benthic-foraging Australian fur seal, *A. p. doriferus* (Kirkman and Arnould, 2018). The study aimed to characterize heart rate profiles in seals during both at-sea and onshore periods and, more broadly, develop a holistic understanding of the sea-to-shore heart rate profile. This approach enabled examination of whether a relationship exists between heart rate while at sea and heart rate while onshore, achieved by quantifying cumulative heartbeats as the area under the heart-rate curve for three broad behavioural states (dive cycles, inter-bout, and onshore), thereby capturing both short-term activity bouts and longer-term sea-to-onshore physiological responses.

## 2 Materials and methods

### 2.1 Study site and devices

Adult female Cape fur seals (CFS) and adult female Australian fur seals (AUFS) that were provisioning pups (lactating and potentially pregnant) were selected for this study, with procedures and research approved by the Animal Ethics Committee of Deakin University (A33/2004) and a DEECA Wildlife Research Permit (10002269). The seal breeding colonies were located at Kleinsee on the northwest coast of South Africa (CFS; -29.677871, 17.070489), and Kanowna Island in south-eastern Australia (AUFS; -39.154359, 146.310549). Seals were captured and data collected between April and August from 2003 to 2008. Seals were selected at random, captured with a modified hoop net (custom built for CFS; Fuhrman Diversified, Flamingo, TX, U.S.A. for AUFS), and anesthetized with isoflurane delivered by portable vaporizer (1 to 5%; Gales and Mattlin, 1998). Once anesthetized, the individuals were removed from the capture net and weighed (± 0.5 kg) on a stretcher using a suspension scale (AUFS) or placed on a platform load-cell balance (CFS).

Each seal was instrumented with (**S1**) [1] a heart rate transmitter (HRX, Wildlife Computers, Redmond, WA, USA) connected to two external electrodes (2.5 cm diameter; Fedak et al., 1983), fixed to the bottom of mushroom-shaped Perspex domes (7.5 cm diameter), at the end of water-proof coaxial cables (each 20–30 cm long); and [2] a heart rate data logger (HTR, Wildlife Computers) which detected the transmitted signal and recorded the instantaneous heart rate at 10 s intervals); [3] an electronic dive behaviour recorder (MK8 or MK10, Wildlife Computers) that sampled dive depth at 5 s intervals; [4] a VHF transmitter (Sirtrack Ltd, Havelock North, New Zealand) to assist in relocating individuals for recapture, and; [5] individual numbered plastic tags (Super Tags^®^, Dalton Supplies, Woolgoolga, N.S.W. 2456, Australia), to aid in individual identification. In total, all devices weighed <0.5% body mass and, consequently, were unlikely to have caused significant additional swim drag (Arnould and Hindell, 2001).

The HRX and electrodes were placed along the dorsal midline with the two electrodes 50 to 70 cm apart (exact distance varied according to animal size), with the anterior electrode located between the scapula, and the HRX in between the electrodes. Two circular patches of fur matching the size of the electrodes were then removed with a pair of scissors and scalpel blade to reveal bare skin. Silicon jelly was applied to each site and quick-setting two-part epoxy (RS Components, Corby, U.K.) rubbed into the fur surrounding it. The electrodes were then put on the skin and the Perspex domes pressed down firmly. As the epoxy hardened, this ensured close contact between the electrodes and skin. The HRX and HRT were placed alongside each other and attached to the dorsal fur using the same epoxy. The HTR displayed the HRX signal received by blinking LED lights such that the quality of the signal and the accuracy of the heart rate recorded could be validated (using a stethoscope) on the animal while it was being handled. The dive recorder and VHF transmitter were also placed along the dorsal midline and attached to the fur with quick-setting epoxy. The individual numbered plastic tags were fitted to the trailing edge of both fore flippers. After devices had been attached, the seal was left to recover from anaesthesia and resume normal behaviour. Individuals were recaptured (as above) after one or more sea-and-onshore cycle.

### 2.2 Data processing

Data from monitoring devices were initially downloaded onto portable computers as hexadecimal files and converted to comma-delimited text files using 3M and Instrument Helper software (both from Wildlife Computers). Time series data from the heart rate and dive behaviour loggers underwent assessment for clock drift over the deployment period and were merged, after which time they underwent inspection in R (version 4.3.0; R Core Team, 2023). Due to detachment of the ECG electrodes from the skin of seals over time, recorded data became gradually erratic and unreliable. Consequently, taking a conservative approach, analyses were restricted to the first sea-and-onshore cycle (time at-sea and subsequent onshore period before returning to sea).

The raw heart rate profiles are shown separately for each species due to the different foraging patterns (Kirkman and Arnould, 2018). Alongside raw heart rate traces, a *moving average* heart rate was calculated using *dplyr::rollmean* (Wickham et al., 2026) with *k* set to 1 hour moving average to simplify visualization of traces of heart rate collected at-sea and onshore. Using just the onshore data, a mean heart rate for every one-hour bin was calculated for each seal, and the minimum of these were selected to show a seal’s *onshore minimum heart rate*. This minimum value was then used to designate the end of the onshore peak. Hereafter, average is used to describe a mean of within-individual means (± SE).

### 2.3 Models

To assess changes in both dive behaviour and heart rate during at-sea foraging trips, two separate linear mixed-effects models were constructed to a subset of bout-only dives (five or more dives in a row). Firstly, to evaluate how dive duration varied within and across dive bouts, a linear mixed-effects model was fitted with fixed effects for dive position within the bout, the total number of dives in the bout, bout number (representing progression through a foraging trip), and species. All two-way interactions among the fixed effects were included to account for potential species- and bout-specific effects on dive duration. Given the strong correlation between the maximum depth of a dive and dive duration (*r* = 0.74), maximum depth of a dive was excluded from the model to prevent multicollinearity. It is noted, however, that maximum depth is an established predictor of dive duration. Individual identity was modelled as a random intercept to account for repeated measures. Secondly, to evaluate how heart rate at the point of maximum dive depth changed throughout dive sequences, a second linear mixed-effects model was fitted. Fixed effects included bout number, number of dives within a bout, species, and all two-way interactions. Individual identity was again included as a random effect. For both models, significance of fixed effects was assessed using Type III Wald chi-square tests. Model performance was evaluated using marginal and conditional *R^2^* values to quantify variance explained by fixed effects alone and by the full model, respectively.

To determine whether a relationship existed within and between heart rate profiles recorded at-sea and onshore, areas over time and under the heart rate curve (cumulative beats·min^-^¹, AUC) were calculated using an outer function with XY inputs and the *trapz* function from the *pracma* package, which applies the trapezoidal rule to estimate definite integrals (Borchers, 2019). Six areas were calculated per individual. Five of the six areas were calculated from heart rate data collected while the seal was at-sea: (1) *dives AUC* within a dive bout and representing a cumulative area of all dives per individual, (2) *surface AUC* within a dive bout and representing a cumulative area of all surface periods between dives per individual, (3) *dive delta AUC* calculated as *surface AUC* minus *dives AUC*, (4) *inter-bout AUC* representing a cumulative area spent on the sea surface between dive bouts (5) *at-sea AUC* represents *dive delta AUC* plus *inter-bout AUC* or the cumulative at-sea area. The final area was (6) *onshore AUC* representing a cumulative area for the onshore period. All six AUC were analysed with simple linear regression to assess heart rate relationships across at-sea activities and between at-sea and onshore periods. Residual plots and Q-Q plots confirmed that model assumptions were reasonably met and resulting *R^2^* and *p* values are presented.

## 3 Results

A total of 545,190 heart rate measurements (beats·min^-^¹, sampled every 10 s) were recorded across complete sea-and-onshore cycles from 12 seals (4 CFS, 8 AUFS) with 382,422 measurements taken at-sea and 162,768 onshore (**Table 1**). Data were obtained from two additional CFS recaptured after their first trip to sea but before they had completed a stay onshore. Average (± SE) sea-and-onshore cycle durations were CFS 5.5 ± 0.9 (2.7, 8.2) AUFS 3.8 ± 0.8 (1.0, 6.9), respectively. Average (± SE) body mass was greater in AUFS (72.0 ± 5.7 kg) than CFS (51.5 ± 2.8 kg).

**Table 1:** Summary table of capture and heart rate metrics for females of two seal species: Cape fur seals (CFS, *N* = 6) and Australian fur seals (AUFS, *N* = 8), all provisioning pups (lactating and potentially pregnant).

Capture date	Spp-ID	Mass (kg)	Trip duration (days)
26/06/2003	AUFS-1	45.5	1.2
27/06/2003	AUFS-2	79.0	1.6
28/06/2003	AUFS-3	93.5	6.9
27/06/2005	AUFS-4	79.5	1.0
06/06/2006	AUFS-5	74.5	4.7
10/06/2006	AUFS-6	52.5	5.4
18/04/2007	AUFS-7	67.0	3.1
18/04/2007	AUFS-8	84.5	6.2
02/07/2006	CFS-1	48.8	8.2
05/07/2006	CFS-2*	42.0	4.7*
06/07/2006	CFS-3	53.0	4.0
22/06/2007	CFS-4	51.8	5.9
23/06/2008	CFS-5*	63.4	2.7*
23/06/2008	CFS-6	50.2	7.7
AUFS		72.0 ± 5.7 (45.5, 93.5)	3.8 ± 0.8 (1.0, 6.9)
CFS		51.5 ± 2.8 (42.0, 63.4)	5.5 ± 0.9 (2.7, 8.2)

The table includes capture date (dd-mm-yyyy), species and ID number (Spp-ID), mass (kg), and duration of one trip (*i.e.*, data used for analysis). The final two rows show mean ± SE (min, max) values per species. The * indicates at-sea profiles only.

### 3.1 At-sea

**Figure 1:**
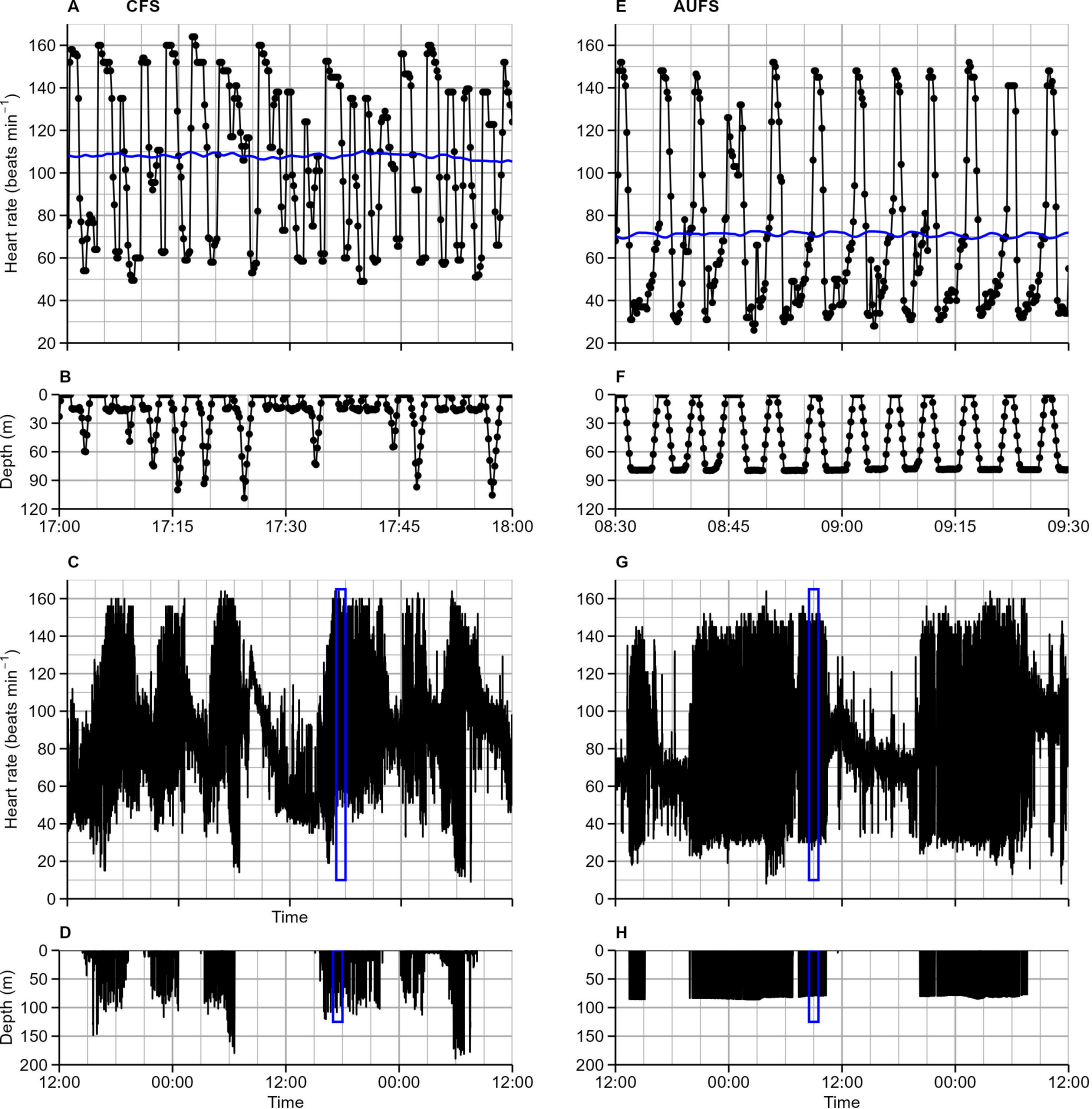
At-sea instantaneous heart rate (beats·min^-1^, **A, C, E, G**) and diving depth (m, **B, D, F, H**) from a Cape fur seal (**A–D**, CFS-1) and Australian fur seal (**E-H**, AUFS-3) over 1 hour **(A, B, E, F)** and a 24-hour period **(C, D, G, H)**. The variable black line and black dots **(A, C, E, G)** show recorded heart rate. Blue boxes in **(C, D, G, H)** show the 1-hour period selected for **(A, B, E, F)**; the blue line in **(A, E)** is a moving average heart rate.

Time spent at sea was on average (± SE) 60.4 ± 14.2 hour and 96.5 ± 13.7 hour (51.8, 142.8), for CFS and AUFS respectively, and much of this time was spent on the sea surface, representing 65.0 ± 3.1% and 63.6 ± 3.2% of total at sea time. Surface periods included inter-dive intervals that were short with high heart rate variability or inter-bout intervals that were longer with more stable heart rates (**Figure 1**, **Table 2**). The remainder of the time spent at sea predominantly involved diving, with distinct dive profiles represented by sequences of bradycardia and tachycardia as seals descended and ascended the water column, typified by greater heart rate variation (**Figure 1**). The average minimum and average maximum (± SE) heart rate throughout the dive sequence was 14.8 ± 1.3 beats·min^-1^ and 161.1 ± 1.5 beats·min^-1^ for CFS; for AUFS, it was 5.7 ± 1.6 beats·min^-1^ and 163.8 ± 2.6 beats·min^-1^. Across the entire at-sea period, including the surface periods and dive sequences, average heart rate was 81.4 ± 1.9 beats·min^-1^ (CFS) and 74.3 ± 1.7 beats·min^-1^ (AUFS) (**Table 2**). As previously described (Arnould and Hindell, 2001; Kirkman et al., 2019) CFS undertook predominantly pelagic dives (68%) while AUFS conducted predominantly benthic dives (72%, **Table 2**).

**Table 2:** Summary table of heart rate profiles for Cape fur seals (CFS) and Australian fur seals (AUFS).

	CFS	AUFS
(A) at-sea (CFS = 6; AUFS = 8)		
total time at-sea (hour)	60.4 ± 14.2 (12.0, 115.6)	96.5 ± 13.7 (51.8, 142.8)
time spent diving (%)	32.6 ± 2.8 (22.3, 42.7)	33.8 ± 3.2 (21.2, 48.4)
time on surface (%)	67.3 ± 2.8 (57.3, 77.7)	66.2 ± 3.2 (51.6, 78.8)
mean heart rate (beats·min^-1^)	81.4 ± 1.9 (72.8, 86.9)	74.3 ± 1.7 (69.0, 78.9)
minimum heart rate (beats·min^-1^)	14.8 ± 1.3 (8.0, 21.0)	5.7 ± 1.6 (1.0, 10.0)
maximum heart rate (beats·min^-1^)	161.1 ± 1.5 (152.0, 164.5)	163.8 ± 2.6 (156.0, 169.0)
benthic diving (%)	31.3 ± 5.4 (18.2, 40.7)	71.5 ± 5.5 (54.1, 97.0)
pelagic diving (%)	68.4 ± 6.0 (48.7, 86.8)	28.5 ± 5.5 (3.0, 45.9)
(B) onshore (CFS = 4; AUFS = 8)		
onshore duration (h)	29.9 ± 6.4 (5.6, 53.9)	53.3 ± 7.1 (43.2, 73.4)
time before *onshore minimum heart rate* (%)	77.7 ± 9.5 (54.6, 98.6)	55.9 ± 11.3 (16.1, 93.8)
mean heart rate (beats·min^-1^)	71.9 ± 1.9 (63.2, 79.7)	60.2 ± 5.0 (47.6, 69.1)
minimum heart rate (beats·min^-1^)	35.7 ± 1.1 (32.0, 41.0)	19.2 ± 6.1 (1.0, 26.0)
maximum heart rate (beats·min^-1^)	133.5 ± 4.4 (113.5, 148.0)	143.3 ± 8.9 (130.2, 169.0)
maximum heart rate at 1^st^ peak (beats·min^-1^)	84.3 ± 4.8 (63.0, 99.0)	81.0 ± 6.1 (67.0 93.0)
*onshore minimum heart rate* (beats·min^-1^)	61.4 ± 2.6 (49.7, 71.5)	41.9 ± 3.6 (33.5, 50.6)
time to *onshore minimum heart rate* (h)	19.4 ± 5.5 (1.2, 39.5)	42.6 ± 9.3 (23.6, 63.4)

Table includes species mean of within individual means (± SE) with individual minimum and maximums. Here, the average is calculated as a mean of within individual means. Metrics are from (A) at-sea and (B) onshore.

**Figure 2:**
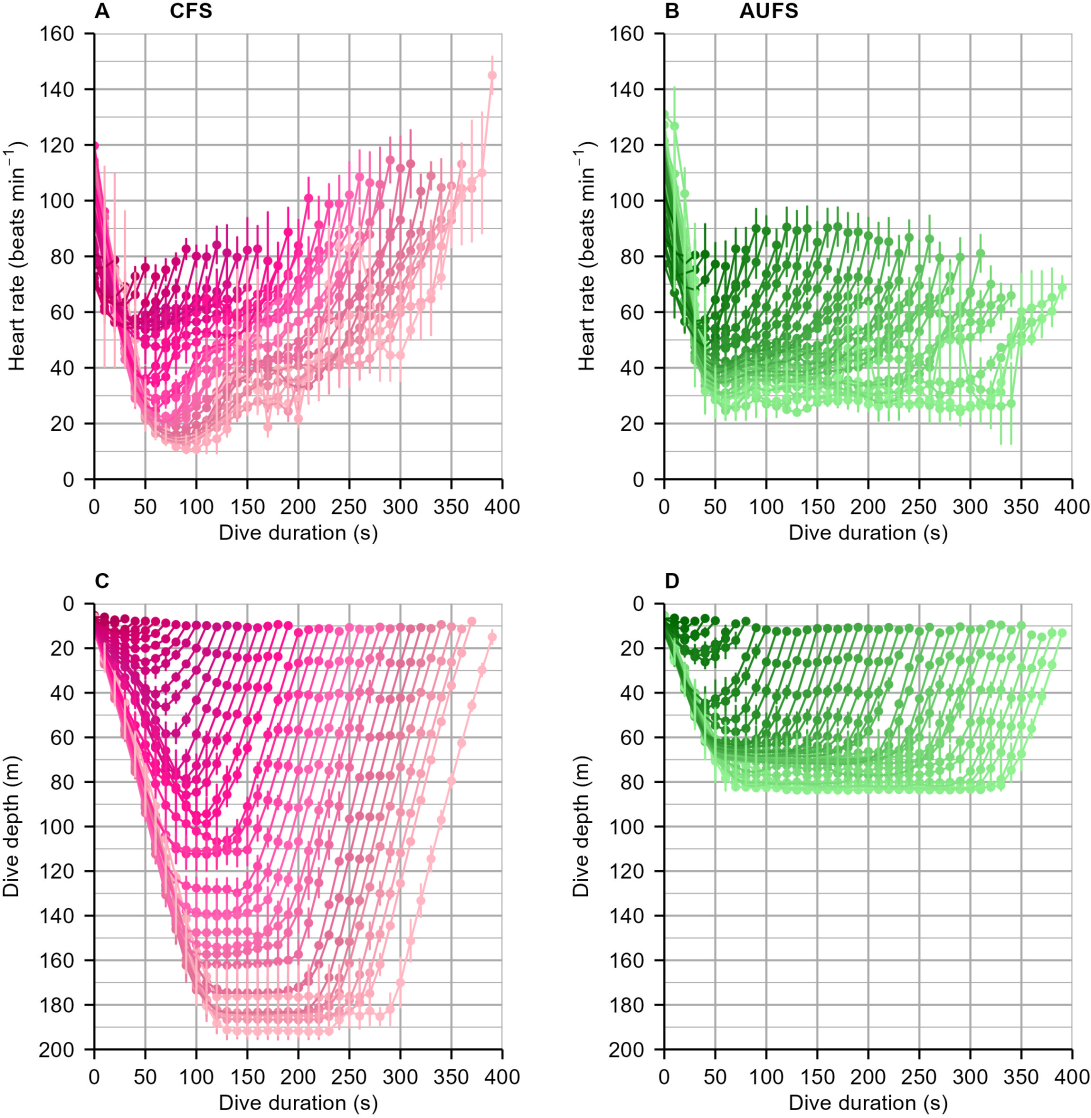
Average heart rate (beats·min^-1^ ± SE, **A, B**), dive depth (m, **C, D**), and duration of dives (s, **A–D**) within a dive bout for Cape fur seals **(A, C)** and Australian fur seals **(B, D)** representing pelagic and benthic foraging modes, respectively. The curves show how heart rate varies during dives over the full range of dive durations; each curve is a line drawn among dives in 10 sec bins ranging from 10 to 400 sec. All dives from all dive bouts (defined as sequences of five or more dives in succession) during the sampling period are included. Points represent the mean of individual means at each bin from the dive start; error bars show ±1 SE across individuals. All data represent measurements taken after the seal initiated a dive (below the sea surface). Elevated heart rates (tachycardia) at the onset of submergence in **(A, B)** are maintained during the initial phase of the dive. Toward the end of submergence, an increase in heart rate is observed before surfacing, especially in Cape fur seals. The shading is to help distinguish dives of differing total duration, with longer dives represented by lighter shading.

The extent and magnitude of bradycardia was related to dive structure, with longer, deeper dives eliciting the greatest reduction in heart rate (**Figure 2**). Compared to the CFS, the AUFS (benthic) maintained less extreme bradycardia and steadier heart rate for extended periods at depth (maximum average dive depth = 83.7 m), often not returning to start-of-dive heart rate levels by the end of the dive. In contrast, CFS (pelagic) reached a lower minimum heart rate that began to increase shortly after the maximum depth of the dive had been reached (maximum average dive depth = 192.0 m), despite individuals remaining at depth; the heart rate appeared to return to start-of-dive levels upon returning to the sea surface.

The best linear mixed effects model for explaining dive duration revealed significant main effects of the number of dives in a bout, bout number, and species, as well as multiple interactions (all *p* < 0.001, **Table 3**). Dive duration decreased with increasing number of dives in a bout (−0.23 s per dive) and with increasing bout number (−1.74 s per bout). Overall, CFS performed shorter dives than AUFS by 89.56 s at the reference levels. However, interactions indicated these effects were species- and context-dependent. For CFS, the negative effect of the number of dives in a bout was stronger (−0.21 s per dive), while the negative effect of bout number was partially offset (1.88 s per bout), resulting in longer dives in later bouts compared to AUFS. Dive position within a bout had a small positive effect for CFS (0.27 s per dive position) versus near-zero for AUFS, and was further influenced, although minimally, by the number of dives in a bout (−0.002 s per dive × dive position) and by bout number (−0.003 s per dive × bout number). A small positive interaction between the number of dives and bout number (0.02 s per dive × bout) suggested a modest compensatory effect across bouts.

**Table 3:** Fixed effects estimate from the linear mixed-effects model predicting dive duration (s) of Cape fur seals and Australian fur seals.

Fixed effects	Est.	SE	DF	*t stat*	*p*
(Intercept)	202.70	11.64	15.21	17.41	< 0.001
number of dives in a bout	-0.18	0.06	4942.00	-3.16	0.00162
bout number	-1.93	0.23	4841.00	-8.39	< 0.001
spp (CFS)	-87.05	17.56	14.43	-4.96	< 0.001
dive position	-0.01	0.06	4949.00	-0.11	0.91208
number of dives in a bout × bout number	0.02	0.00	4954.00	6.83	< 0.001
number of dives in a bout × spp (CFS)	-0.21	0.05	4933.00	-4.02	< 0.001
bout number × spp (CFS)	1.89	0.25	4725.00	7.40	< 0.001
number of dives in a bout × dive position	0.00	0.00	4949.00	-3.57	< 0.001
spp (CFS) × dive position	0.27	0.06	4949.00	4.31	< 0.001
Random effects	Variance	SD			
id	937.2	30.61			
residual	4067.4	63.78			

This model shows the effects of the number of dives in a bout, the bout number, species (a factor), dive positions, and interactions among these predictors: number of dives × bout number, number of dives × species, bout number × species, number of dives × dive position, and species × dive position. Individual ID was included as a random effect to account for repeated measures (*R^2^_m_*= 0.17; *R^2^_c_*= 0.33; *N* = 14; *n* = 4971).

A separate linear mixed-effects model examining heart rate at maximum dive depth revealed significant effects of the number of dives in a bout, bout number, species, and their interactions (all *p* < 0.05, **Table 4**). Heart rate at maximum depth increased slightly with the number of dives in a bout, with each additional dive associated with a 0.03 beats·min^-1^ increase. A modest increase in heart rate across successive bouts was also observed, with each additional bout associated with a 0.21 beats·min^-1^ increase. Notably, CFS exhibited lower heart rates at maximum depth than AUFS overall (by 72.27 beats·min^-1^ at the reference levels), and these species differences were further modified by interactions. For CFS, heart rate at maximum depth increased more steeply across bouts (by an additional 0.59 beats·min^-1^ per bout) and decreased with increasing dive count within a bout (by 0.11 beats·min^-1^ per dive).

**Table 4:** Fixed effects estimate from the linear mixed-effects model predicting heart rate (beats·min^-1^) at the maximum depth of each dive within dive bouts in Cape fur seals and Australian fur seals.

Fixed effects	Est.	SE	DF	*t stat*	*p*
(intercept)	109.44	4.88	12.78	22.43	< 0.001
bout number	0.21	0.08	4870.07	2.62	0.009
spp (CFS)	-31.98	7.49	12.98	-4.27	0.001
number of dives in a bout	0.03	0.01	4953.44	2.22	0.027
bout number × spp (CFS)	0.59	0.09	4867.56	6.26	< 0.001
spp (CFS) × number of dives in a bout	-0.11	0.02	4954.80	-6.10	< 0.001
Random effects	Variance	SD			
id	181.7	13.48			
residual	551.9	23.49			

This model shows the effects of bout number, species (a factor), number of dives in a bout, and interactions among these predictors: bout number × species, and species × number of dives in a bout. Individual ID was included as a random effect to account for repeated measures (*R^2^_m_*= 0.22; *R^2^_c_* = 0.42; *N* = 14; *n* = 4971).

### 3.2 Onshore

**Figure 3:**
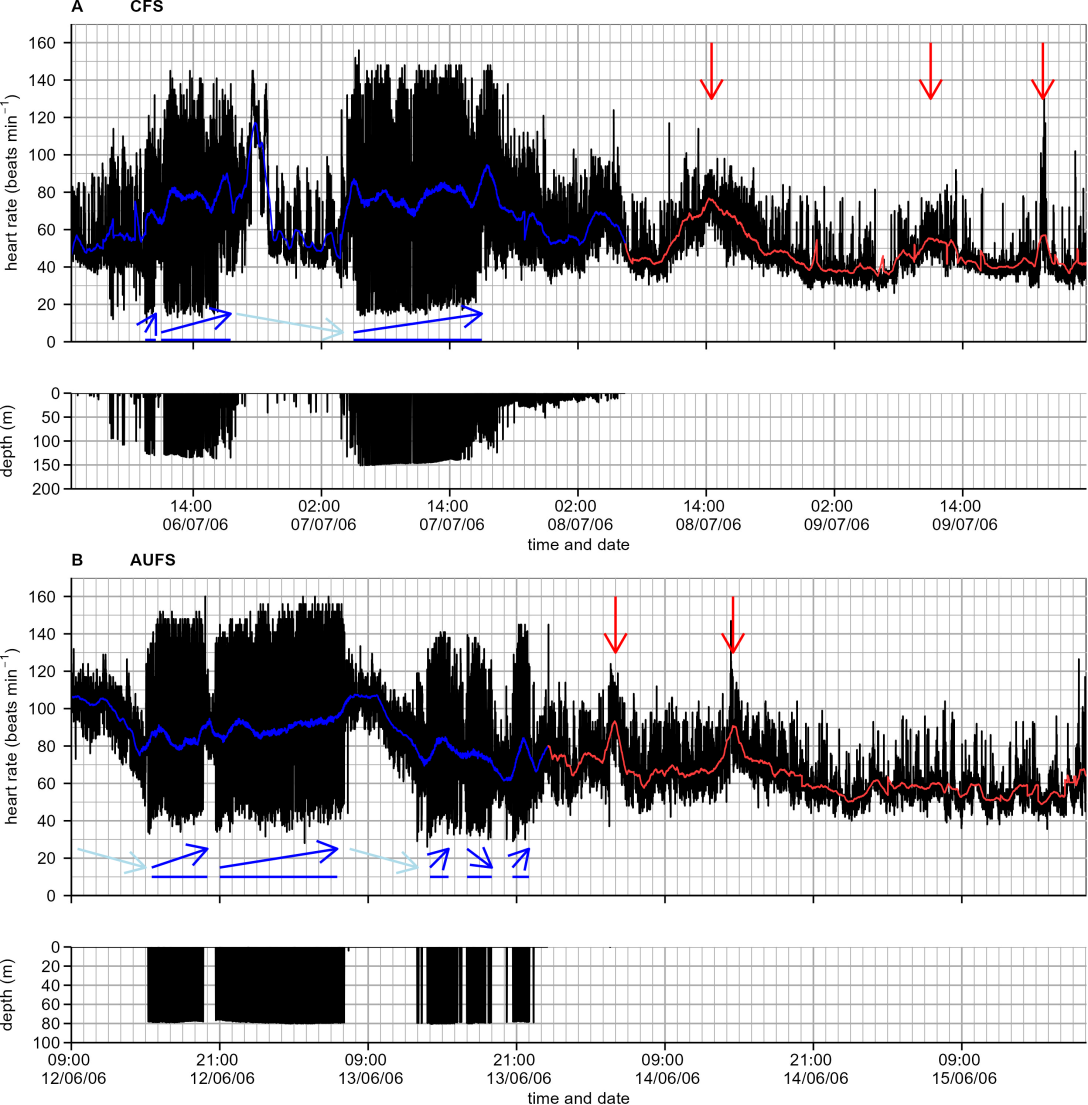
Instantaneous heart rate (beats·min^-1^) while at-sea and onshore, and diving depth from a **(A)** Cape fur seal (CFS-2) and **(B)** Australian fur seal (AUFS-6) over several days. The variable black line shows heart rate or depth, while the blue at-sea and red onshore variable lines indicate a moving average heart rate. Blue arrows indicate general heart rate trend; dark blue is diving periods, light blue indicates that the seal was on the sea surface, and red arrows indicate onshore peaks in heart rate.

Following the foraging trip to sea, seals returned and remained onshore for, on average (± SE), 29.9 ± 6.4 h (CFS) and 53.3 ± 7.1 h (AUFS). Given what is known about the activities undertaken by fur seals while onshore (*i.e.*, predominantly rest), a relatively flat and otherwise low heart rate compared to sea profiles was expected. In part, a low heart rate was reflected in the average onshore heart rate (± SE) of 71.9 ± 1.9 (CFS) and 60.2 ± 5.0 beats·min^-1^(AUFS). However, a large portion of the onshore heart rate profile fluctuated rather than being flat and beyond what might be expected of apnoea and eupnoea cycles, typically rising to unexpectedly high levels. In some individuals, heart rate was already elevated upon return from the sea and increased further, while in others, heart rate began low and gradually rose (**Figure 3**). Notably, each onshore heart rate profile often contained multiple heart rate peaks, with the first peak often being the most pronounced, followed by smaller secondary and tertiary peaks. The first of the onshore heart rate peaks reached, on average (± SE), 81.0 ± 6.1 beats·min^-1^ for CFS, 7.8 ± 3.2 h after individuals had hauled out, and 84.3 ± 4.8 beats·min^-1^ for AUFS, 6.0 ± 1.7 h after individuals had returned to land. Following these heart rate peaks, heart rate decreased to similar levels observed during diving bradycardia at-sea (**Figure 3**), with seals eventually reaching an *onshore minimum heart rate* of 61.4 ± 2.6 beats·min^-1^ (CFS) and 41.9 ± 3.6 beats·min^-1^ (AUFS). However, these minimums were not reached until 19.4 ± 5.5 h (CFS) and 42.6 ± 9.3 h (AUFS) after hauling out and, accordingly, seals spent 77.7 ± 9.5% (CFS) and 55.9 ± 11.3% (AUFS) of their onshore time with a heart rate elevated above their *onshore minimum heart rate*.

### 3.3 At-sea and onshore heart rate relationship

**Figure 4:**
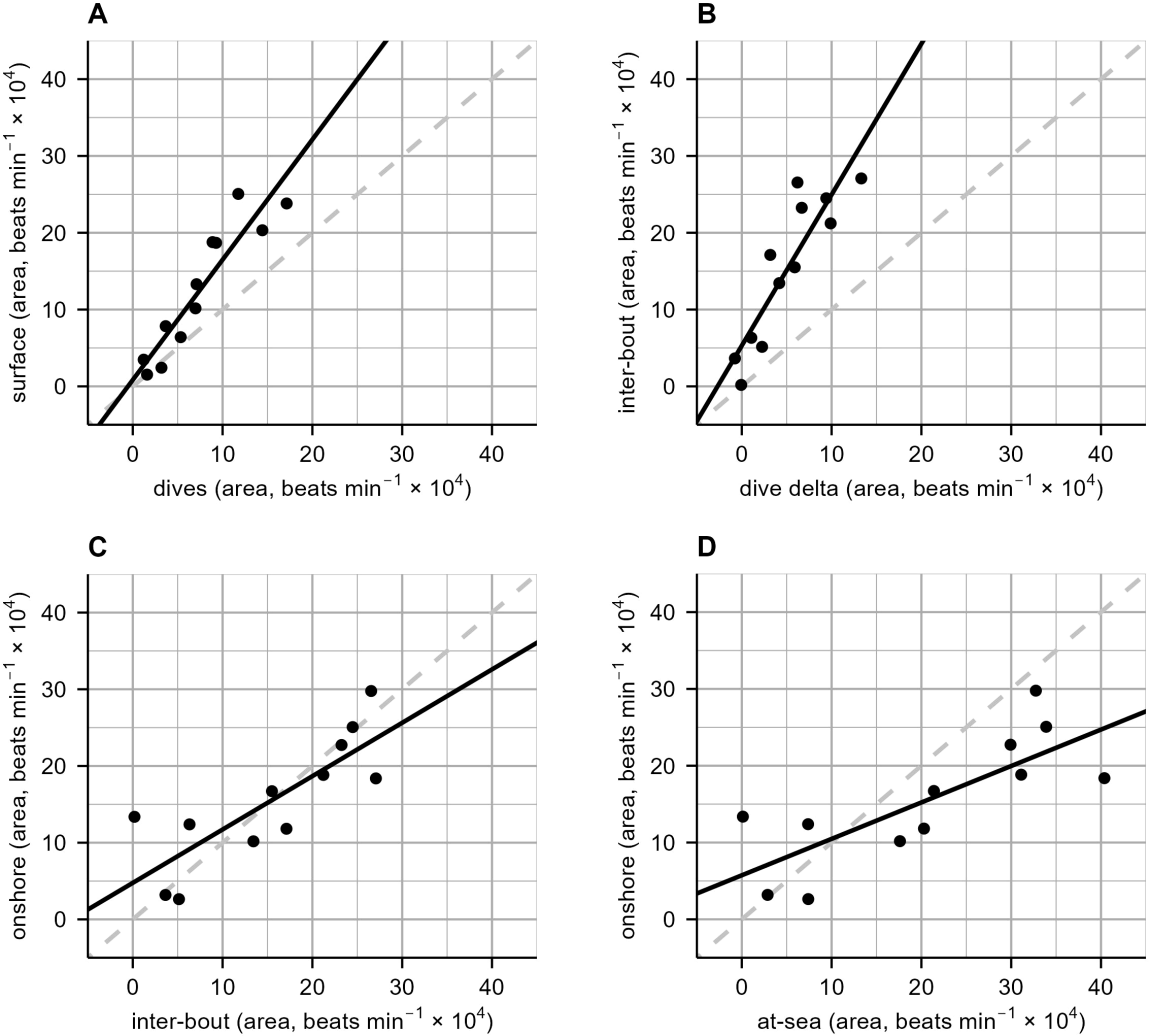
Four linear regressions among and between at-sea and onshore area under the heart rate curve (cumulative beats·min^-1^), from Cape fur seals and Australian fur seals. Area was calculated using time and the heart rate curve. The black solid line represents the linear relationship, and the grey dashed line represents where X is equal to Y. Each black point represents an individual. For plots **(A, B)**, Cape fur seals and Australian fur seals are *N* = 6 and *N* = 8, while plots **(C, D)**, are *N* = 4 and *N* = 8 respectively; fewer data were collected from Cape fur seals onshore. Model outputs are presented in **Table 5**.

To investigate whether any relationships existed within and between heart rate maintained at-sea and onshore, six areas under the heart rate curve (cumulative beats·min^-1^) were compared. Given that both species showed a similar and unexpected increase in heart rate while onshore, and given their otherwise close taxonomic relatedness, data for both species were pooled for AUC analysis. A very strong positive and significant relationship was observed between *dives AUC* and the preceding *surface AUC* (*p* < 0.001), explaining 86% of the variance, with an estimated slope of 1.56 beats·min^-1^ per unit increase in surface area, indicating that seals maintained a greater heart rate tachycardia area on the surface between dives compared to the area of bradycardia during dives (**Figure 4a**, **Table 5a**). The relationship between *dive delta AUC* (area representing *dives AUC* minus *surface AUC*) and the area representing *inter-bouts AUC* was a strong positive and significant relationship (*p* < 0.001; **Figure 4b**, **Table 5b**), explaining 78% of the variance, with an estimated slope of 1.97 beats·min^-1^ per unit increase in inter-bout area. As *dive delta AUC* increased, *inter-bout AUC* also increased. The relationship between *inter-bout AUC* and that of all *onshore AUC* (**Figure 4c**, **Table 5c**) was strong, positive, and significant (*p* = 0.001), explaining 65% of the variance with an estimated slope of 0.70 beats·min^-1^ per unit increase in onshore area, indicating that as the *inter-bout AUC* increased, so too would *onshore AUC*. Lastly, the relationship between *at-sea AUC* (representing *dive delta AUC* plus *inter-bout AUC*) an *onshore AUC* was significant, strong and positive (*p* = 0.003; **Figure 4d**, **Table 5d**), explaining 61% of the variance with an estimated slope of 0.47 beats·min^-1^ per unit increase in onshore area. This indicated that an increase in the *AUC* representing all surface periods at-sea while accounting for diving costs would reflect an increase in the *AUC* onshore.

**Table 5:** Summary of linear regression analysis conducted to explore the relationships between regions of heart rate area (cumulative beats·min^-1^, AUC) for Cape fur seals and Australian fur seals at-sea and onshore across four models.

Model	Term	Est.	SE	*95% CI*	*t* stat	*p*
A	(intercept)	8378.19	18231.08	-32243.20, 48999.58	0.46	0.656
	*dives AUC* × *surface AUC*	1.56	0.20	1.11, 2.02	7.68	< 0.001
		residual SE = 33902, *R^2^* = 0.86, *R^2^_adj_* = 0.84, F_1,12_ = 58.97				
B	(intercept)	53003.93	21445.51	5220.35, 100787.50	2.47	0.033
	*dive delta AUC* × *inter-bout AUC*	1.97	0.33	1.24, 2.69	6.01	< 0.001
		residual SE = 46644, *R^2^* = 0.78, *R^2^_adj_* = 0.76, F_1,12_ = 36.08				
C	(intercept)	47603.22	28550.25	-16010.71, 111217.10	1.67	0.126
	*inter-bout AUC* × *onshore AUC*	0.70	0.16	0.34, 1.05	4.35	0.001
		residual SE = 50658, *R^2^* = 0.65, *R^2^_adj_* = 0.62, F_1,10_ = 18.91				
D	(intercept)	57338.44	29118.99	-7542.71, 122219.60	1.97	0.077
	*at-sea AUC* × *onshore AUC*	0.47	0.12	0.21, 0.74	3.94	0.003
		residual SE = 53933, *R^2^* = 0.61, *R^2^_adj_* = 0.57, F_1,10_ = 15.50				

For (A) and (B), Cape fur seals and Australian fur seals are *N* = 6 and *N* = 8, while (C) and (D), are *N* = 4 and *N* = 8 respectively; fewer data were collected from Cape fur seals onshore. Results are visualized in **Figure 4** and are labelled correspondingly: A, B, C, D.

## 4 Discussion

**Figure 5:**
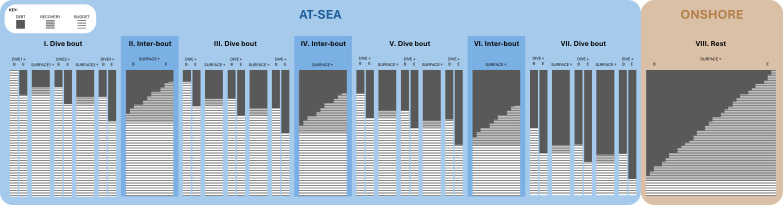
Conceptual framework illustrating how heart rate reflects debt (grey), recovery (grey striped) and budget (white striped) managed by Cape fur seals and Australian fur seals while at sea (blue) and onshore (brown). 1. Dive bout **(I)** represents a period when a seal is actively foraging at sea. (↓) At the start of the first dive bout, a seal begins with a full budget; however, debt accrues between the beginning (B) and end (E) of the first dive (DIVE1). The present study discusses how dive debt reflects lactic acid and nitrogen accumulation, oxygen depletion, and changes in gaseous exchange when dives exceed the aerobic dive limit. (↑) Brief surface intervals between dives (SURFACE1) enable partial recovery through active modulation. The present study discusses how seals use these short surface periods to rapidly repay some oxygen debt, clear lactic acid, and initiate anaerobic and/or metabolic suppression recovery. However, these periods do not fully clear accrued debt. As seals begin the next dive (DIVE2), the debt setpoint increases and the available budget decreases. Although subsequent surface periods (SURFACE2) provide partial recovery, additional dives (e.g., DIVE3) continue to accrue debt. 2. Inter-bout **(II)** represents a prolonged period of swimming at the sea surface between dive bouts. At the beginning (B) of the inter-bout, seals retain the maximum debt accumulated during the preceding dive bout. During this period, seals appear to clear some of this debt, which the present study considers in relation to gas exchange, digestive processes, and physiological stabilization of heart rate. However, because seals must prioritize continued foraging (i.e., initiating the next dive bout), not all debt may be cleared by the end (E) of the inter-bout. Consequently, although the inter-bout facilitates partial recovery, the debt setpoint increases and the available budget decreases, reflected at the start of the subsequent dive bout **(III)**. 3. Successive dive bouts **(III, V, VII)** continue to accrue additional debt despite intervening inter-bout periods **(IV, VI)** that permit partial debt clearance. As a result, by the end of the seals’ time at sea **(VII)**, they carry a maximum cumulative debt. 4. Rest **(VIII)** represents the onshore haul-out period. Seals arrive onshore carrying maximum debt that must be cleared between the beginning (B) and end (E) of this period. The present study proposes that elevated heart rates observed onshore reflect processes involved in clearing debt accrued at sea. Additionally, the present study explores other factors that may contribute to elevated onshore heart rate, including travel exertion, digestion, moulting, lactation, foetal growth, environmental conditions, and behavioural responses to those conditions.

A holistic examination of heart rate profiles in Cape fur seals (CFS) and Australian fur seals (AUFS) revealed a pronounced heart rate peak when the seals were onshore. This peak occurred 6 h (AUFS) to 7.8 h (CFS) post-arrival, reaching 81 to 84 beats·min^-1^, and offsetting the time it took for seals to reach an '*onshore minimum heart rate*' by 19.4 to 42.6 hours. While at-sea heart rate recordings were largely consistent with what was anticipated from previous research (detailed below), the presence of these large, unexpected onshore heart rate peaks suggests that otariid physiological recovery from foraging may be more protracted, complex and occur over much longer timescales than previously understood. These results also reinforce existing literature (detailed below) indicating that, while seals haul out to rest among other behaviours, this “restful” period may reflect a complex interplay between behavioural motivations and physiological adjustments. The subsequent discussion, aided by a conceptual framework (**Figure 5**), will explore the likely multifaceted explanation for this onshore heart rate peak, exploring potential deferred at-sea physiological costs while considering distinct onshore energetic demands.

### 4.1 Heart rate at sea

Dive duration declined both within and across dive bouts, potentially reflecting accumulating physiological constraints such as rising metabolic demand, depleted oxygen reserves, or incomplete recovery. In parallel, heart rate at maximum dive depth tended to increase across bouts, particularly in CFS, suggesting a reduced capacity to sustain pronounced bradycardia over the course of foraging trips. While heart rate adjustments during dive bouts, the sequences of dives, and surface intervals support those found in past studies (Elsner et al., 1964; Irving et al., 1941; McDonald and Ponganis, 2014; Scholander, 1940; Thompson and Fedak, 1993), area under the heart rate curve (cumulative beats·min^-^¹, AUC) analysis in the present study suggests that these patterns may reflect not only active modulation during foraging but also attempts to offset physiological debt accumulated over the course of dive bouts. Specifically, a very strong positive relationship was found between *dives AUC* and corresponding *surface AUC*, indicating that seals maintain higher heart rate on the surface immediately following a dive compared to the heart rate during the preceding dive, suggesting short-term compensation. This response is consistent with previously described surface periods that facilitate rapid oxygen perfusion (Ponganis, 2011). Similarly, Costa and Valenzuela-Toro (2021) reported that in sea lion species, individuals performing longer dives exhibit longer post-dive surface intervals; in this instance dives of up to 7 minutes and surfacing of up to 15 minutes were considered.

Elevated *surface AUC* relative to *dives AUC* likely reflects a combination of mechanisms governing cardiovascular recovery following dives. One interpretation is that the seals are employing anaerobic metabolism during dives, thereby requiring increased cardiovascular effort at the surface to repay oxygen debt or clear lactic acid, a scenario consistent with species known to exceed their estimated aerobic dive limit (ADL) in the field (Costa et al., 2001). An alternative (or complementary) interpretation is that the animals extend dives by metabolic suppression achieved via bradycardia, and that the elevated *surface AUC* reflects a rapid tachycardic response to restore circulatory flow and re-establish aerobic metabolism, without substantial reliance on anaerobic pathways (Fedak and Thompson, 1993). As the AUC metric integrates heart rate over time rather than directly measuring oxygen debt or lactate accumulation, the analysis cannot discriminate definitively between these mechanisms. Though, given that otariid seals frequently exceed their ADL (Costa et al., 2001), and that extended post-dive surface intervals commonly follow long dives (as above), the observed patterns are most consistent with anaerobic recovery, although metabolic suppression likely plays a contributory role (Davis, 2014).

While it appears that the immediate post-dive surface periods provide opportunities for physiological recovery, this study also considered the longer inter-bout intervals, where seals remain at the sea surface for extended durations without diving. These “rest” periods at sea also appear to play a role in physiological balance; here, a strong positive relationship between *dive delta AUC* and subsequent *inter-bout AUC* was found. This suggests that longer periods on the sea surface, between dive bouts, may serve as a form of latent compensation for particularly demanding or inefficiently compensated dives. Such findings indicate that even while “resting” at the sea-surface, seals may still be actively managing physiological states resulting from their previous underwater exertions. Previously, this period has been described as a time used to facilitate rapid gas exchange (Ponganis, 2011). Williams et al. (2004) observed that oxygen recovery (ml O_2_ kg^–1^) at the sea surface was associated with the number of strokes in a dive, dive duration and type of food captured, while Sparling et al. (2007), observed it as a time to prioritize oxygen-dependent processes like digestion. While gas exchange and digestive processes are likely to contribute to the initial post-bout elevation in heart rate, this period may also represent a time for physiological stabilization, during which accumulated dive-related debt is gradually addressed. This is reflected in the eventual decline of heart rate to lower thresholds (as in this study), potentially enabling the initiation of subsequent dive bouts. Collectively, patterns within and across dive bouts suggest that repeated diving may incur a physiological debt that is not fully alleviated during short surface intervals at-sea, whether between individual dives or during longer pauses between bouts. Full recovery may instead require more prolonged rest periods, such as those spent onshore.

### 4.2 Impact of at-sea activities on onshore heart rate

The heart rate modulation during diving and periods at the sea surface appears to contribute to at-sea recovery. However, these dynamics do not fully account for the elevated heart rate recorded following haul-out. A strong positive relationship between the *dive delta AUC* and subsequent *inter-bout AUC* indicates that particularly demanding or inadequately compensated dive bouts are followed by extended recovery at the surface. Moreover, correlations between *at-sea AUC* and *onshore AUC* reveal potential lingering effects of dive effort, with dive effort cumulating over several days and recovered within the first portion of the seals’ return to land. Elevated heart rate onshore may therefore reflect carryover effects of dive effort as seals recover from extended foraging bouts. Accordingly, in this study, an even stronger correlation emerges when considering the *onshore AUC* and cumulative *at-sea AUC*. Such patterns imply that the physiological strain accumulated during foraging may persist into the post-haul-out period and may require additional time onshore for full recovery.

One physiological challenge at sea that could contribute to such deferred effects is the management of respiratory gases and metabolic byproducts, particularly when foraging involves dives approaching or exceeding ADL. The dive response involves significant heart rate reduction and peripheral vasoconstriction to conserve oxygen (Kaczmarek et al., 2018). If a seal’s dives are long enough to surpass its ADL, an increase in anaerobic metabolism can lead to lactic acid accumulation, necessitating extended surface time for clearance (Fedak and Thompson, 1993; Kooyman et al., 1980). Additionally, gases like nitrogen dissolve into the blood during deeper dives and can only be safely offloaded at the sea surface, once gas exchange is restored. As a result, seals appear to face the challenge of managing rather than minimizing nitrogen load while at-sea (Hooker et al., 2012), presumably to balance metabolic risks with the need to maximize foraging opportunities. In the present study, the *surface AUC* was typically greater than the *dives AUC*; it is conceivable that with an increasing number of dives within a bout, perfusion to some tissues might become insufficient for complete recovery between every dive. Fur seals often operate near or exceed their ADL (Costa et al., 2001). Cape fur seals have a behavioural ADL of 3.85 min (Kirkman et al., 2019) and Australian fur seals have a calculated ADL of 1.7 min (Costa et al., 2004). In this study, recorded dives reached up to 5.8 mins, meaning both seals frequently exceed their ADL. Consequently, lactic acid, nitrogen, or other metabolic byproducts may have accumulated. If these were not fully cleared during the at-sea portions of the foraging cycle, even by means of reduction in dive duration and extended surface time, and perhaps due to competing needs like continued foraging or initial stages of digestion (Sparling et al., 2007), the debt might be deferred, potentially contributing to the elevated heart rate patterns observed once the seals were onshore.

### 4.3 Heart rate onshore

Upon hauling out after foraging at-sea, heart rate profiles of both seal species revealed an unexpected peak. Seals exhibited a pronounced peak in heart rate from 6 h (AUFS) to 7.8 h (CFS) after arrival, reaching an average of 81 (CFS) to 84 (AUFS) beats·min^-^¹. It was only after these peaks that *onshore minimum heart rate* was reached, on average ~42 beats·min^-^¹ (AUFS) to ~61 beats·min^-^¹ (CFS) and approaching heart rates reflective of REM sleep (as low as 35 beats·min^-^¹, see Lyamin and Siegel, 2019). These unexpected post-arrival heart rate peaks delayed seals from reaching this *onshore minimum heart rate* by 19.4 h (CFS) to 42.6 h (AUFS).

Longer term data on heart rate after hauling out may provide new clues as to the recovery mechanisms being carried out. The onshore phase for pinnipeds is generally considered a time for rest, where any fluctuation in heart rate is expected to align with apnoea and eupnoea cycles associated with sleep, circadian or behavioural thermoregulation, and potentially the terrestrial practice of dive response components, *i.e.*, cycling through apnoea and eupnoea as part of their breath-hold development (De León et al., 2024; Deacon and Arnould, 2009; Willis et al., 2025). Consequently, while some variability in heart rate was anticipated (De León et al., 2024; Lin et al., 1972), a generally stable heart rate pattern over time was the prevailing expectation. However, findings of the present study did not support this. Although elevated onshore heart rate may result from physiological debt accrued during at-sea diving, it is important to recognize that other factors are also driving variation in heart rate and can complicate analysis and interpretation, as discussed below.

### 4.4 Impact of onshore activities on onshore heart rate

One possible explanation for the onshore peak heart rate could be the recovery from the exertion of traveling between distant foraging sites and the colony. Fur seals operate as central-place foragers, often facing considerable food competition within the range of their breeding colony (Jeanniard-du-Dot and Guinet, 2021). The lactating Australian fur seals in this study, for example, can travel distances up to 320 km (Kirkwood and Arnould, 2012). Such journeys impose significant energetic demands, not only from diving but also from the sustained effort of swimming back to the colony. This is especially true for lactating fur seals who may increase trip durations to maximize energy intake (Costa, 2007; McHuron et al., 2019), and for any seal, as surface travel can involve evasive manoeuvres to avoid predation (Shaughnessy, 2006). Thus, the peak in heart rate observed onshore might, in part, reflect the physiological recovery from this cumulative travel-related physical exertion.

The onshore peak heart rate may also be related to metabolic demands of digesting a substantial meal, a process known to increase metabolic rate (MR) and, consequently, heart rate (Green, 2011; Secor, 2009). While seals are known to digest some food at-sea (Sparling et al., 2007), any prey obtained towards the end of a foraging trip or opportunistically en route to the colony would likely be processed onshore. Given that digestion and assimilation can take 8 to 12 hours before a fasting state is reached (Davis and Davis, 2019), the timing of the heart rate peaks observed in this study (6 and 8 hours post-arrival) shows a temporal correspondence that might suggest a link. The peak heart rate could, therefore, be related to the energy expended on the digestion, absorption, and assimilation of food, which typically involves substantial increases in MR (Secor, 2009). The delayed timing of digestive activity may therefore represent an ecological advantage, allowing seals to prioritize foraging while at sea and to process prey and recover energetically onshore, when exposure to predation risk is reduced. Support for this interpretation comes from previous studies on pinnipeds that have documented postprandial MR peaks. For example, onshore-resting juvenile Steller sea lions exhibited a peak 3.7 hours after feeding, lasting up to 10 hours (Rosen and Trites, 1997). Similarly, postprandial MR increased within 30 minutes post-feeding in swimming juvenile harbour seals, *P. vitulina*, and remained elevated for up to 12 hours (Markussen et al., 1994). Sparling et al. (2007) also noted MR spikes in juvenile and adult grey seals, *H. grypus*, 0 to 11 hours after feeding during extended sea-surface intervals (termed ‘inter-bout’ in this study), suggesting these ‘payback’ periods were deferred from foraging dives. Considering these examples, it is plausible that the seals in the present study also initiated significant digestive processes upon hauling out, contributing to the observed heart rate elevation.

Two other considerations underpinned by metabolic demand were considered. The first was the energetic cost of moulting. Previous studies have shown that resting metabolism (and therefore heart rate) typically rises during moult in otariids (Ladds et al., 2017). This elevation is likely due to the direct energetic cost of pelage replacement, the need to raise body surface temperatures to support tissue regrowth, or a combination of both factors. However, data in this study were collected outside of moult for AUFS that moult between February and March (Austral summer and autumn) (Lynch et al., 2011), but likely with some overlap with CFS moult that is more staggered, starting in February (summer) and finishing towards the end of June (winter; pers. obs.). While moult may contribute to the elevated heart rate onshore and should be studied further, its effect was not captured in this study for AUFS and may be difficult to detect due to the prolonged moulting period of CFS. Secondly, the onshore peak heart rate could reflect other shifts in energy demand, particularly for the lactating and potentially pregnant CFS and AUFS from which data were collected. These individuals spend a significant portion of their time budget “resting” onshore (Ladds et al., 2018), yet specific physiological processes such as milk production and foetal growth persist during these periods. On the one hand, foetal growth is known to increase heart rate in humans and presumedly seals too, reflecting increases in energy demand and processing to grow offspring (Hunter and Robson, 1992). That said, several studies have suggested that milk production by otariids involves a minimal direct metabolic cost (Costa and Gentry, 1986; Costa and Trillmich, 1988), and, in humans and presumedly seals, lactation and the act of suckling activate neurohormonal pathways that help maintain or reduce heart rate, promoting maternal calm and facilitating efficient milk transfer, rather than increasing cardiovascular workload (Groer et al., 2013). These points argue that while pregnancy costs may increase heart rate, lactation may not be a major contributor to increased heart rate in hauled-out females; however, more research on this topic is required.

Local climatic conditions were also considered as a potential influence on the peak heart rate observed onshore. De León et al. (2024) found that South American sea lions, *Otaria flavescens*, resting onshore showed higher average heart rate in warmer ambient temperatures (21 to 25 °C) compared to cooler conditions (0 to 5 °C). However, as data in the present study were collected from April to July (autumn and winter in the Southern Hemisphere) warmer seasonal conditions were not captured. As such, while temperature-related effects on heart rate cannot be ruled out, they cannot be explored further without dedicated investigation across a broader range of environmental conditions. In considering other environmental influences, it was noted that climatic conditions may interact with the substrate on which fur seals rest and impact the posture they adopt (Willis et al., 2025); movements made *in situ* or between resting locations in response to environmental changes could contribute to changes in heart rate, though again, this would require further investigation. Finally, the physical effort involved in movements and coming onshore might have contributed to the increase in heart rate. While the seals in this study typically moved 30 to 100 meters inland across gently sloping sandy beaches (CFS) or up the rocky slope to the top of Kanowna Island (AUFS), they did so in less than 20 minutes at a gentle pace (pers. obs.). However, as the increase to peak heart rate occurred gradually over approximately 7 hours, this initial locomotory effort, or even *in-situ* movements and postural adjustments, are unlikely to be major factors in the prolonged elevation in heart rate.

### 4.5 Conclusions

The present study suggests that elevated onshore heart reflects a physiological adaptation to recover from the deferred costs of foraging at sea while meeting ongoing energetic demands onshore. Contributing factors likely include prioritizing feeding and digestion, though additional, as yet unidentified, processes may also play a role. An additional strength of this study is that the heart rate data were originally collected over a decade ago. These long-term, archived datasets allow for the application of modern analyses to reveal insights that were not apparent at the time of collection, and similar datasets from other species could be reanalysed to advance understanding of physiological regulation without re-instrumenting animals. Future studies could quantify the relative contributions of these factors, for example by linking dive effort, foraging success, and digestive status to onshore cardiac patterns, to clarify the mechanisms of the physiological response.

This study highlights the value of analysing long-term heart rate profiles in wild mammals. By examining continuous data rather than isolated short-term intervals, it becomes possible to uncover both immediate and carry-over physiological responses that influence individual condition over time. Monitoring behaviours over extended periods is essential to link short-term mechanism to longer term outcomes, providing data that can inform ecological and energetic models, and support management and conservation decision-making. Such holistic approaches also raises broader questions, for example, what truly constitutes a “resting” heart rate under natural conditions. This work serves as a foundation for future research investigating the drivers of onshore cardiac responses in seals, and more broadly, for adopting long-term physiological monitoring to advance an integrated understanding of heart rate regulation in wild mammals.

## Data Availability

The summary data supporting the conclusions of this article will be made available by the authors, without undue reservation.

## Supplementary material

SUPPLEMENTARY FIGURE 1 [1] a heart rate transmitter connected to two external electrodes, [2] a heart rate data logger, [3] an electronic dive behaviour recorder, [4] a VHF transmitter and, [5] individual numbered plastic tags.
