# Supplementary figures and images for: Aquatic and terrestrial heart rates in fur seals: evidence for delayed metabolic processing
Source: Front Physiol. 2026 Apr 30;17:1755942. doi: 10.3389/fphys.2026.1755942 (PMC13171587; doi:10.3389/fphys.2026.1755942)

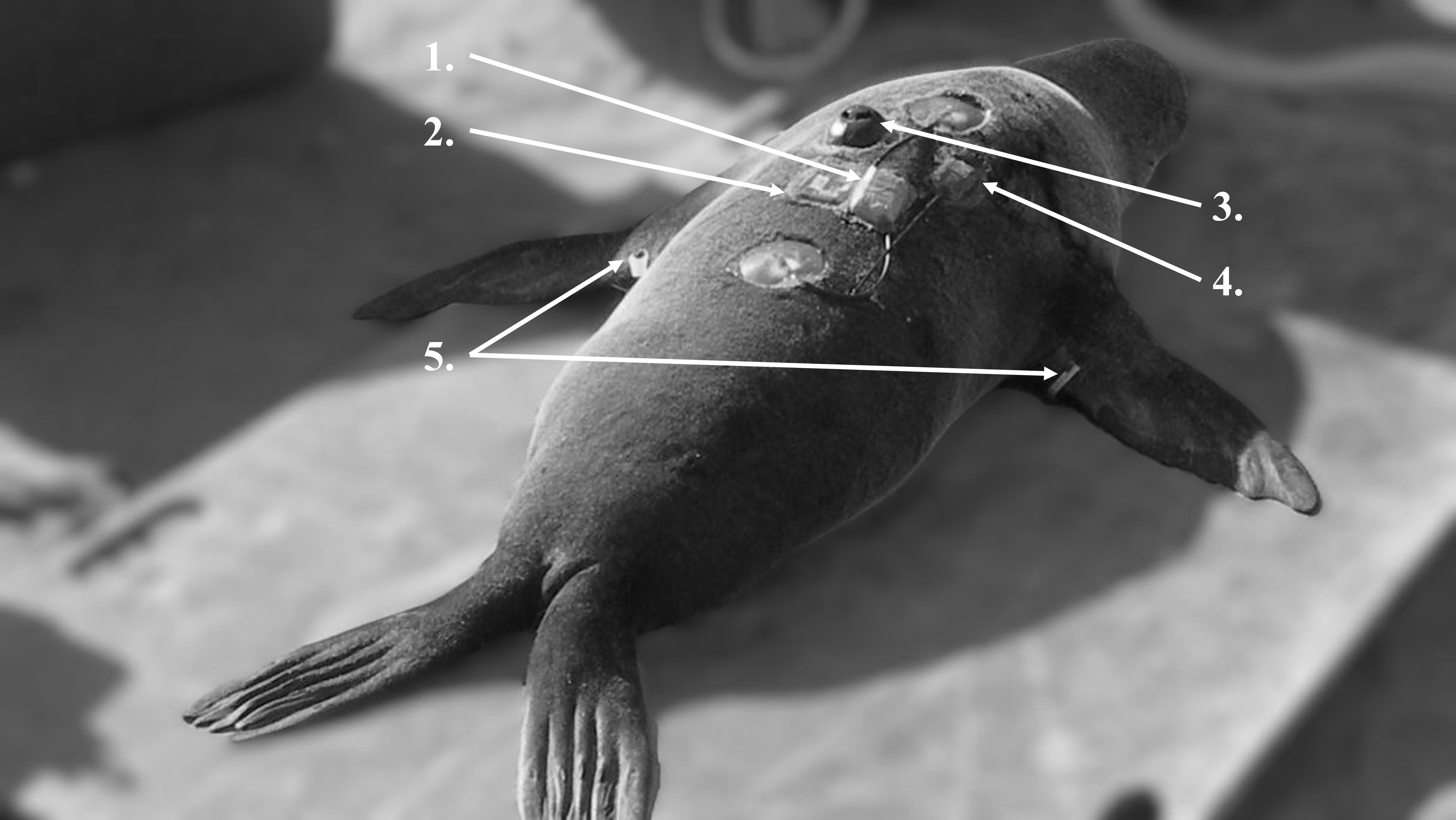

Supplement: SUPPLEMENTARY FIGURE 1 — [1] a heart rate transmitter connected to two external electrodes, [2] a heart rate data logger, [3] an electronic dive behaviour recorder, [4] a VHF transmitter and, [5] individual numbered plastic tags. [file Image1.png]
